# The prevalence of underweight in children aged 5 years and younger attending primary health care clinics in the Mangaung area, Free State

**DOI:** 10.4102/phcfm.v10i1.1476

**Published:** 2018-05-28

**Authors:** Danae Koetaan, Andrea Smith, Anke Liebenberg, Marietjie Brits, Christos Halkas, Maresa van Lill, Gina Joubert

**Affiliations:** 1 School of Medicine, University of the Free State, South Africa; 2 Department of Family Medicine, University of the Free State, South Africa; 3 Department of Biostatistics, University of the Free State, South Africa

## Abstract

**Background:**

The Constitution of South Africa stipulates that all children have the right to basic nutrition; however, a great number of South African children are underweight for age. It is important to address malnutrition as it is associated with more than 50% of all child deaths in developing countries and also increases the risk for infective diseases.

**Aim:**

To determine the prevalence of underweight in children aged 5 years and younger attending primary health care clinics in the Mangaung area, Free State, and determine the possible underlying causes thereof.

**Setting:**

Six preselected primary health care clinics in the Mangaung area.

**Methods:**

This was a cross-sectional study. Demographic and clinical information and anthropometric measurements were collected from the children’s Road-to-Health clinic cards, obtained from the children’s caregivers.

**Results:**

In total, 240 children were included, of which 51.7% were girls. The median age was 7.5 months. The weight-for-age graph revealed that 7.7% (95% confidence interval: 4.8%; 11.9%) of children were underweight or severely underweight for age. Length-for-age and weight-for-height graphs were mostly incomplete. Underweight children differed from normal weight children regarding birth weight (low birth weight 70.6% vs. 12.4%) and history of malnutrition (60.0% vs. 7.1%).

**Conclusion:**

The prevalence of underweight in children aged 0–5 years attending primary health care clinics in Mangaung is 7.7% based on information available from Road-to-Health cards. This figure could be higher if these cards were filled in more accurately. A low birth weight and history of malnutrition are associated with underweight.

## Introduction

In 1948, the United Nations Children’s Fund (UNICEF) declared freedom from hunger and malnutrition a basic human right.^1^ However, malnutrition remains a huge problem. In 2011, an estimated 101 million children under 5 years were underweight and 165 million had stunted growth.^2^ Recently, UNICEF included child malnutrition in its Millennium Development Goals for 1990–2015 with the aim to reduce the mortality rate of children under 5 years by two-thirds.^3^ In 2015, the global under-5 mortality rate had decreased by 53%.^4^ According to a report by UNICEF in 2016, stunting also declined from 39.6% to 23.2% worldwide.^5^ In 2013, South Africa had 1.3 million children under the age of 9 years who were underweight and 2.3 million with stunted growth.^6^ Between 2009 and 2013, all provinces in South Africa, except the Free State (3.9% in 2009 to 10.7% in 2013), were able to reduce the incidence of severe malnutrition. However, the incidence of malnutrition is still higher than the national target of 10 per 1000 children under the age of 5. In 2011, the Free State also had the highest under-5 mortality rate (72.1 per 1000 live births) compared with the country’s mortality rate of 38.5.^7^

More than 50% of all child deaths are associated with malnutrition. In developing countries, infectious diseases and malnutrition are closely associated.^8^ Malnutrition contributes to more infections as a result of low immunity, and infections cause more malnutrition because of higher energy demands. According to UNICEF, the major causes of malnutrition are inadequate food intake and illness, with a vicious cycle of one sustaining the other.^9^ Non-exclusive and suboptimum breastfeeding that takes place in the first 6 months of life results in 1.4 million deaths globally and accounts for 10% of the disease burden occurring in children younger than 5 years.^10^

According to a study by Vorster et al.,^2^ 57% of people in South Africa live in poverty. They noted an association between poverty and well-being, with poverty being both a cause and consequence of malnutrition. Furthermore, 26.5% of children living in rural, poverty-stricken areas are stunted compared to 16.7% of children living in urban areas.

Clinical evaluation and anthropometric measurements are used to assess malnutrition. Clinical signs include visible wasting and general oedema. For anthropometric measurements, the weight, height and mid-upper arm circumference (MUAC) are measured and plotted for specific ages and gender groups. The use of MUAC has been found to be a more accurate measure of nutritional status of a child than the weight-for-height graph.^11^

### Aim and objectives

The aim of this study was to determine the prevalence of underweight in children under the age of five years attending primary health care clinics in the Mangaung area and to determine the possible underlying causes.

## Methods

### Study design

This was a cross-sectional study.

### Setting

Public primary health care clinics in the Mangaung area, driven by professional nurses, deliver comprehensive services, including preventative, chronic and acute medicine. Based on factors such as expected number of children at the clinics and socio-economic area of the clinic, and logistics such as distance to travel to the clinic and time constraints, six primary health care clinics (out of 23 clinics in Mangaung) were purposively selected to take part in the study.

### Study population and sampling strategy

The target population included children aged 5 years and younger attending public primary health care facilities in the Mangaung area. Participating clinics were contacted and preferable weekdays for data collection were set for each clinic based on the number of children usually present on these days. The sample size was set at 240 for statistical and logistical considerations. Clinics were visited once a week during the study period and consecutive patients were sampled until the target sample size for each clinic was met (ranging from 20 to 60, depending on the days of the week the clinics indicated as preferred days for data collection, and the approximate number of children visiting the clinic on these days). Children aged 5 years and younger attending the participating clinics for any reason and who had a clinic card (Road-to-Health card) were included in the study.

Malnutrition includes both overweight (*z*-score > 2 standard deviation [SD]) and underweight (*z*-score < -2 SD); however, for the purpose of this article, the researchers focused on underweight for age. The case definition for underweight for age used was a *z*-score of between -2 and -3 SD and severe underweight for age as a *z*-score of < -3 SD.

### Data collection

A data collection tool was used to extract information from the Road-to-Health card. Variables included weight and head circumference at birth, vaccination status, supplements given, human immunodeficiency virus (HIV) status of mother and child, tuberculosis (TB) status of child, and anthropometric measurements and growth trends. For the pilot study, three Road-to-Health cards were obtained from each of the six selected clinics. No changes were made to the data collection sheet. The pilot study confirmed that the method of data collection, by immediately completing the data sheets once the mother or guardian gave consent, was applicable. Data from the pilot study were included in the analysis as no changes were made to the data sheet.

Data were collected between July and November 2014 by a student researcher. Mothers or guardians in the waiting room at the clinics were approached by a student researcher and asked to participate in the study. Once the study was explained to the mother or guardian, willing participants signed consent forms.

### Data analysis

The student researchers coded the data. Data were analysed by the Department of Biostatistics, Faculty of Health Sciences, University of the Free State. Results were summarised by frequencies and percentages (categorical variables) or medians and percentiles (numerical variables, because of skew distribution).

### Ethical considerations

The protocol was approved by the Ethics Committee of the Faculty of Health Sciences, University of the Free State (STUD NR 19/2014). Permission to conduct the study was obtained from the Free State Department of Health and the participating clinics. No identifying information was captured on the data sheets. By signing the informed consent form, the mother or guardian gave permission for the student researchers to record data from the child’s Road-to-Health card.

## Results

The study consisted of 240 participants of whom 51.7% (*n* = 124) were girls and 48.3% (*n* = 116) were boys. Their median age was 7.5 months (interquartile range [IQR] 3 months, 20 months). Most of the children (*n* = 199/236, 84.3%) had a normal birth weight of 2.5 kg or more. More than 70% of the children were born at 37 weeks or more (*n* = 147/201, 73.1%). The median gestational age was 39 weeks (IQR 23 weeks, 42 weeks). The median head circumference at birth was 34 cm (IQR 32 cm, 35 cm). Almost all the children were exclusively breastfed (*n* = 207/222, 93.2%) during their first 3 months, 5.4% (*n* = 12) were on formula while 1.4% (*n* = 3) received both breast milk and formula.

Of the 240 children, 85.8% (*n* = 206) were up to date with all their vaccinations as required. Thirty children missed more than one immunisation. The most frequently missed immunisations in this group were pneumococcal conjugate vaccines (PCV, 47.6%), measles (40.0%) and hepatitis B (26.7%). Of the 137 children older than 6 months, 79.6% (*n* = 109) had received their 6-month vitamin A supplement. Only 42.9% (*n* = 39) of the 91 children older than 1 year had received their 1-year deworming treatment.

The HIV status of 54 (22.5%) mothers and 72 (30.0%) children was not recorded. Of those with results, 48 (20.0%) mothers and 21 (8.7%) children tested HIV positive. Co-trimoxazole was given to 17 of the 21 HIV-positive children. Most of the 240 children tested negative for TB (*n* = 164, 68.3%).

**FIGURE 1:**
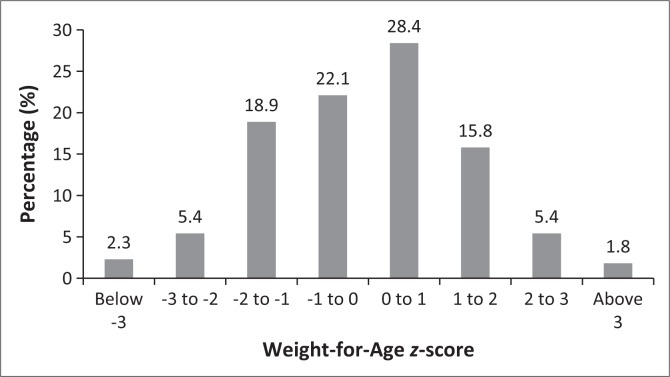
Weight-for-age scores (*n* = 221).

According to the most recent weight-for-age *z*-scores (Figure 1) of the 221 children with sufficient data, only 7.7% (95% confidence interval [CI]: 4.8%; 11.9%) (*n* = 17) were underweight or severely underweight for age (5.4% underweight for age and 2.3% severely underweight for age). There were insufficient recorded data for length for age (only 20.4% recorded), weight for height (only 2.9% recorded) and MUAC (only 11.7% of children aged 6 months and older recorded).

Of the 211 participants with sufficient data, 10.4% (*n* = 22) had a history of previous malnutrition recorded in the Road-to-Health cards, of whom only two were referred for further treatment to a doctor.

There were significant associations between birth weight and a history of previous malnutrition and the current nutritional status (Table 1). Most of the underweight-for-age children (70.6%) had a low birth weight compared to 12.4% of children who had normal weight for age. Sixty per cent of underweight children had a history of previous malnutrition compared to 7.1% of the normal weight children.

**TABLE 1:** Investigated associations with current nutritional status.

Variable	Underweight for age		Normal weight for age		*p*-value
	*n*	%	*n*	%	
**Birth weight**					< 0.0001
< 2500 g	12	70.6	25	12.4	
> 2500 g	5	29.4	177	87.6	
**Total**	**17**	**100.0**	**202**	**100.0**	
**History of malnutrition**					< 0.0001
Yes	9	60.0	13	7.1	
No	6	40.0	169	92.9	
**Total**	**15**	**100.0**	**182**	**100.0**	
**HIV status of mother**					0.1775
Positive	5	29.4	38	18.5	
Negative	7	41.2	122	59.5	
Unknown	5	29.4	45	22.0	
**Total**	**17**	**100.0**	**205**	**100.0**	
**HIV status of child**					0.7217
Positive	2	11.8	16	7.8	
Negative	10	58.8	130	63.4	
Unknown	5	29.4	59	28.8	
**Total**	**17**	**100.0**	**205**	**100.0**	
**Vaccination up to date**					0.4785
Yes	16	94.1	175	85.4	
No	1	5.9	30	14.6	
**Total**	**17**	**100.0**	**205**	**100.0**	
**Vitamin A supplement received**†					1.0000
Yes	6	85.7	103	81.1	
No	1	14.3	24	18.9	
**Total**	**7**	**100.0**	**127**	**100.0**	
**Deworming supplement received**‡					0.6918
Yes	2	33.3	37	44.6	
No	4	66.7	46	55.4	
**Total**	**6**	**100.0**	**83**	**100.0**	

HIV, human immunodeficiency virus.

† , at 6 months of age;

‡ , at 1 year of age.

Vaccination status, HIV status of mother and child, vitamin A and deworming supplement were not significantly associated with current underweight.

## Discussion

According to the weight-for-age graph, 7.7% of children aged 5 years and younger in Mangaung are currently underweight for age. A study by Sambu and Hall^13^ showed that 4.8% of children in South Africa under the age of 5 had evidence of wasting. In 2014, 10.1% of children in the Free State lived in households where there was reported child hunger.^14^

A comparison between birth weight and underweight revealed that 70.6% of the underweight-for-age children had a low birth weight. With an average or an above-average birth weight, the likelihood of developing malnutrition and disabilities later in life may be less.^14^

The vast majority of children were exclusively breastfed, which is considered protective against malnutrition. In the past, formula feeding was encouraged in an attempt to reduce HIV transmission. However, it became apparent that poor sanitation and expensive formula feeds contributed to malnutrition. In 2009, the World Health Organisation (WHO) introduced the Breastfeeding Promotion Programme, which encourages breastfeeding in spite of an HIV-positive status, if antiretrovirals (ARVs) are administered to both mother and child during this time.^15^

Presently, 85.8% of the study population’s vaccinations are up to date. This is lower than the 90.4% reported by the Department of Health in 2009 for children in the Free State under the age of 1 year.^16^ The most common vaccines not administered were for PCV, measles and hepatitis B. Specifically PCV is problematic as malnourished children are more prone to develop respiratory tract infections.^8^ In an attempt to address missed immunisations, the Expanded Program on Immunization has implemented a structured regimen for immunisations as well as catch-up immunisations, which all nurses and doctors are expected to follow.^17^

For 20.4% of the children older than 6 months, there was no evidence that they received their scheduled vitamin A supplement. A study done by Mayo-Wilson et al.^18^ found that vitamin A decreased the rate of mortality and illness in children by 24%. Their study also showed that children who did not receive vitamin A had an increased incidence of diarrhoea and measles. There was also no evidence of deworming treatment for 60% of the children older than a year. This makes them vulnerable to parasitic infections that are detrimental to their state of nutrition and can lead to complications such as anaemia.

In 2010, WHO released revised guidelines on preventing mother-to-child transmission of HIV.^19^ It was predicted that these guidelines would reduce the vertical transmission rate to < 2% in the non-breastfeeding population and to < 5% in the breastfeeding population. A study^20^ by the Human Sciences Research Council in 2008 showed that less than 3% of children aged 2–14 years were HIV positive. In 2012, the HIV prevalence in this group was 1.7%.^21^ This is in contrast with our results which showed that 8.7% of the children were HIV positive. Of these, the majority were given Co-trimaxole. A comparison between the HIV status of the mother and underweight child revealed that 29.4% of currently malnourished children have a HIV-positive mother. It is also alarming that 30% of children’s HIV statuses were unknown, as HIV testing should be done routinely for all children attending primary health care clinics as they qualify for ARV treatment that can prevent further health complications.

The MUAC is the international indicator of malnutrition,^11^ yet only 11.7% of children in our study had their measurements recorded. Acute malnutrition is reflected in the weight-for-height graph and chronic malnutrition and stunted growth are depicted by the length-for-age and height-for-age graphs.

A study^22^ in 2008 showed that 17% of children were stunted and that the prevalence of stunting was 12.2% higher than that for wasting. In our study, 79.6% of the length-for-age graphs and 97.1% of the weight-for-age graphs were not completed. The fact that most of the children’s length-for-age graphs are not completed could indicate that many children with stunted growth are not being identified by the system, especially seeing that presently only the weight-for-age graphs are adequately completed.

### Study limitations

The student researchers were not familiar with the older Road-to-Health card; therefore, it took longer to obtain data from it. The MUAC, length and head circumference were very rarely recorded; the pilot study was too small to pick this up. An unpublished study^12^ done on the prevalence of undernutrition among children (6–59 months) in early childhood development centres in Mangaung concluded that none of the children was undernourished according to their MUAC measurements. The number of children at the clinics was less than that expected, forcing the research team to abandon any form of random sampling and reduce the sample size.

### Recommendations

Future studies should include both children who attend clinics and children whose parents do not bring them to clinics to have a better picture of the incidence of malnutrition in children living in the Mangaung area. Studies should also include a larger study population and more clinics in the Mangaung area to ensure that a representative study population is selected.

The HIV and TB status cannot be recorded as ‘unknown’ in a child’s Road-to-Health card. A definite positive or negative test result should be recorded. These important conditions require a diagnosis to identify children for ARV treatment and early TB treatment to lower the risk of future complications. A rapid HIV test and four-question screening test for TB should be part of standard care.

More emphasis needs to be placed on the administration of vitamin A as well as the recording thereof in the Road-to-Health card. Weight for age, length for age and weight for height should be completed by health care workers during the child’s routine check-ups to ensure all forms of malnutrition and growth deficiency are detected. Measuring and recording of the head circumference is important to ensure that proper brain development is occurring during child growth. Clinical notes need to be recorded by health care workers in the correct section of Road-to-Health card to ensure efficient routine check-ups. Timely vaccination administration, as well as catch-up immunisations should be emphasised to health care workers and parents to protect children from preventable life-threatening diseases and complications. Potential malnutrition can be addressed by preventing low birth weight through proper antenatal care.

## Conclusion

The extent of underweight for age in children aged 5 years and younger who attend primary health care clinics in Mangaung is 7.7% (95% CI: 4.8%; 11.9%). Possible underlying causes may include a low birth weight and history of malnutrition. The lack of recording critical anthropometric measurements (length for age, weight for height, head circumference and MUAC) presents as an indirect cause for undiagnosed malnutrition and associated growth problems in children.
